# Acute suppurative appendicitis associated with *Enterobius vermicularis*: an incidental finding or a causative agent? A case report

**DOI:** 10.1186/s13104-017-2822-3

**Published:** 2017-10-06

**Authors:** Boubacar Efared, Gabrielle Atsame-Ebang, Boubacar Marou Soumana, Layla Tahiri, Nawal Hammas, Hinde El Fatemi, Laila Chbani

**Affiliations:** 1 Department of Pathology, Hassan II University Hospital, Fès, Morocco; 2 Department of Parasitology, Hassan II University Hospital, Fès, Morocco; 3 Faculty of Medicine and Pharmacology, Sidi Mohamed Ben Abdellah University, Fès, Morocco

**Keywords:** *Enterobius vermicularis*, Appendicitis, Diagnosis, Pathology

## Abstract

**Background:**

Histological acute appendicitis patterns associated with *Enterobius vermicularis* is an extremely rare finding. The exact role of this parasite in acute appendicitis is controversial as usually resected specimens show no evidence of histological inflammation.

**Case presentation:**

We present herein a case of a 21-year-old male Arabic patient who presented with clinical syndrome of acute appendicitis. Emergency appendectomy was performed and the histopathological examination of the resected specimen showed the presence of *E. vermicularis* as well as intense acute inflammatory patterns such as mucosal ulceration and suppurative necrosis. The post-operative course was uneventful and the patient was discharged with appropriate anti-helmintic drug prescription.

**Conclusion:**

Acute appendicitis due to *E. vermicularis* is a very rare occurrence. The histopathological analysis of resected specimens should pay special attention to search for this parasite for adequate post-operative treatment of patients.

## Background

Acute appendicitis is the most common abdominal emergency worldwide [1, 2]. Several pathological mechanisms leading to appendiceal wall inflammation and luminal obstruction are thought to be responsible for the disease [3]. *Enterobius vermicularis* or pinworm is one of the most common parasite in humans affecting mainly children, as approximately 4–38% of pediatric population is affected in certain areas of the world [4, 5]. Humans get infected by the fecal–oral route by ingesting the parasite’s eggs from contaminated foods or water [5]. The disease is often asymptomatic but can sometimes manifest classically as nocturnal anal pruritus especially in children. However, cases of acute appendicitis associated with *E. enterobius* have been reported in the literature [6–9]. But, the role of this parasite as a causative agent still remains a subject of speculations, as usually resected specimens show little or no histological signs of inflammation [1, 3]. We describe herein, a case of a young adult patient presenting with acute suppurative appendicitis associated with *E. vermicularis* on histological examination of the resected appendiceal specimen.

## Case presentation

**Fig. 1:**
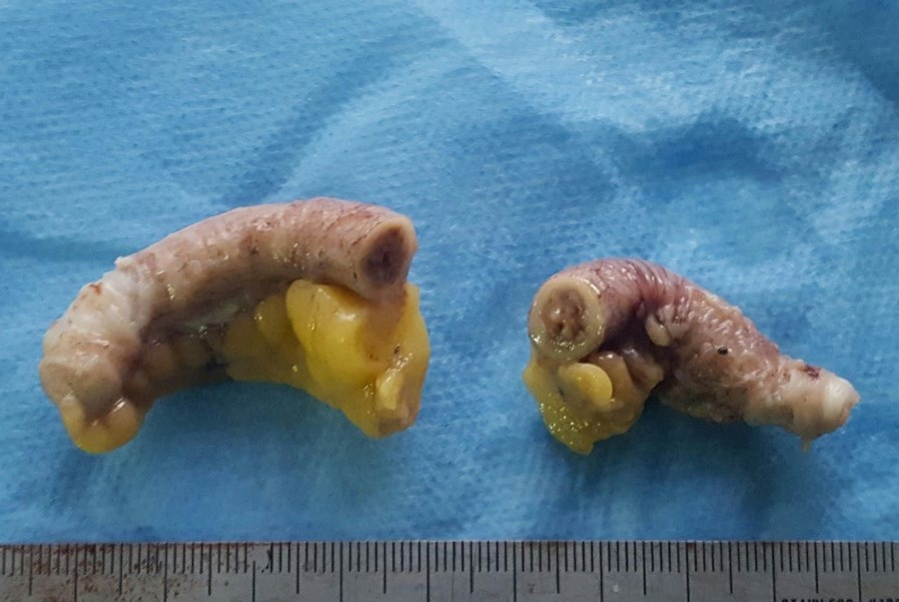
The resected appendix shows an inflamed wall with a lumen filled of thick faeces and stercolith

**Fig. 2:**
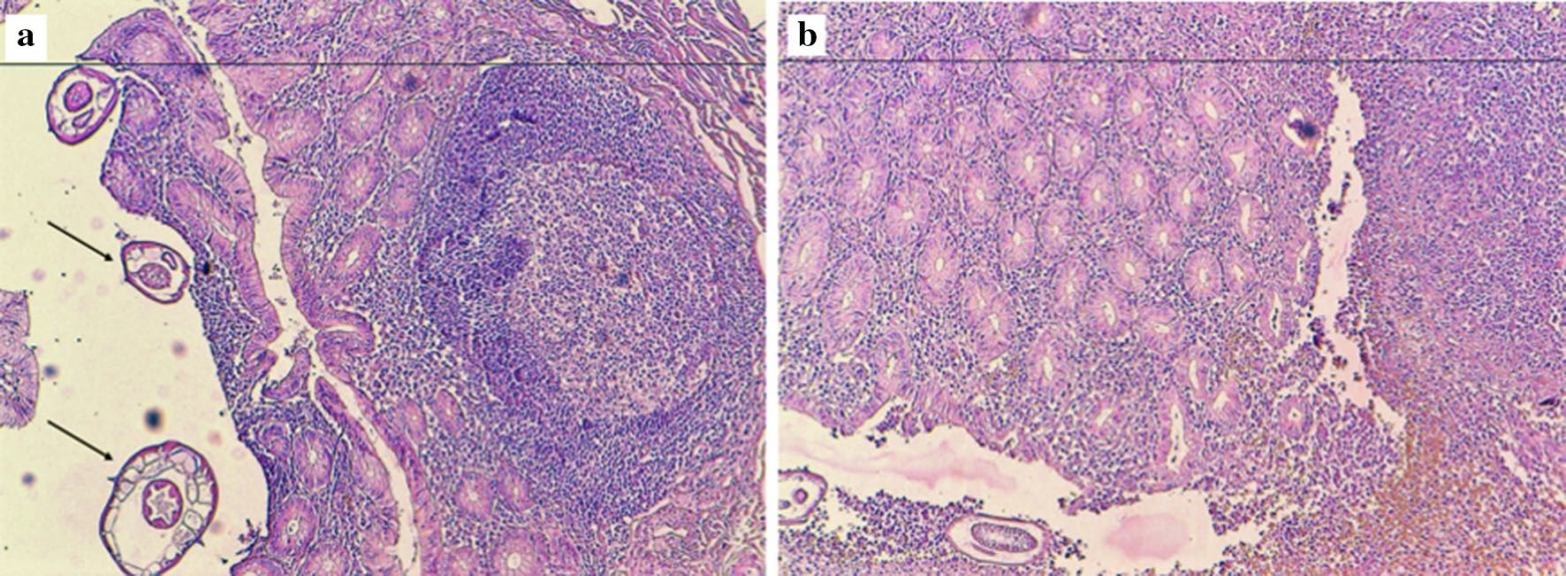
**a** The histological view showing the inflamed appendiceal wall, with parasites in the lumen (black arrows). A hyperplastic lymphoid follicle is also seen in the mucosa and submucosa (hematoxylin–eosin-saffron × 100). **b** Another section of the appendix showing histological signs of intense acute inflammation: epithelial ulceration and suppurative necrosis (hematoxylin–eosin-saffron × 100)

**Fig. 3:**
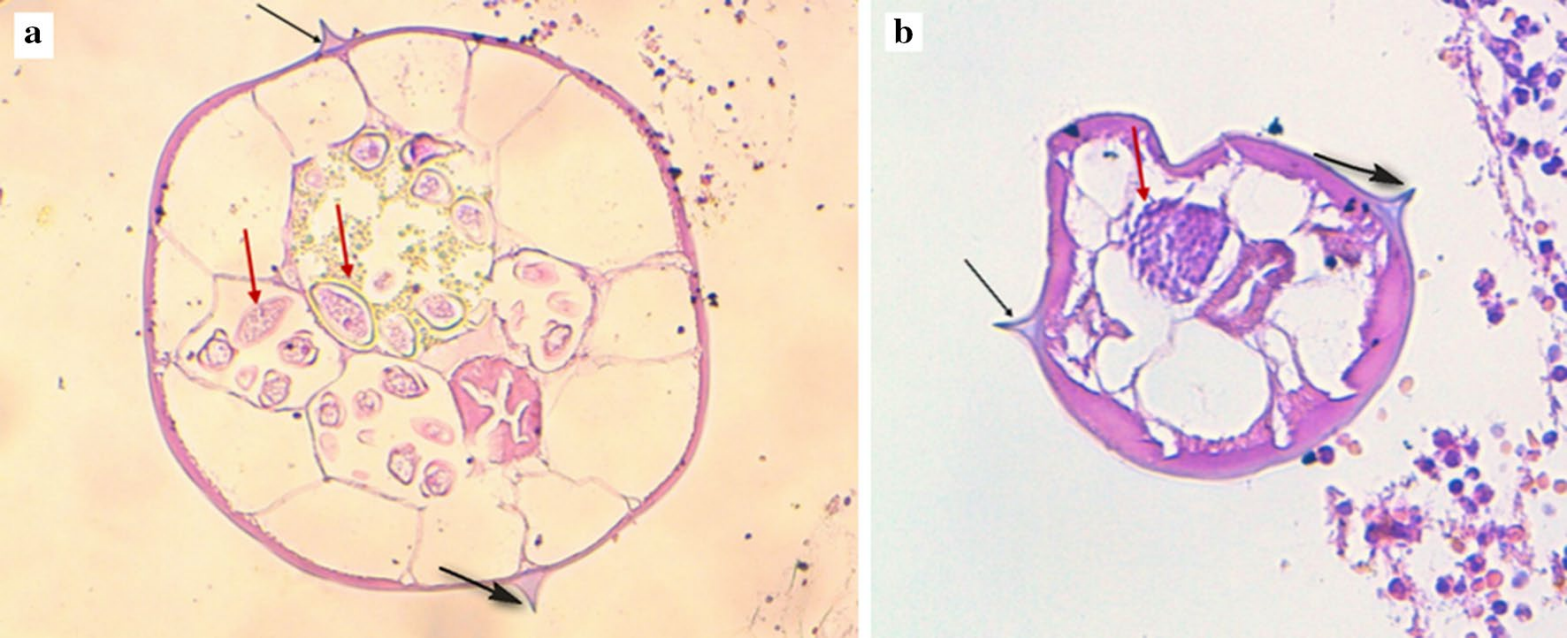
**a** A gravid female parasite with two lateral characteristic alae (black arrows) and uterus filled with many eggs (red arrows) (hematoxylin–eosin-saffron × 400). **b** Histological view of the male parasite with its testicular structures (red arrow) and two lateral alae (black arrows) (hematoxylin–eosin-saffron × 400)

A 21-year-old male Arabic patient was admitted at emergency department for abdominal pain and nauseas for 2 days. Physical examination revealed right iliac fossa pain and tenderness associated with peritonism. The Rovsing’s sign was positive. The patient reported episodes of anorexia, vomiting and a mild fever. Laboratory examination showed a mild increase in white blood count and an increased C-reactive protein. These clinical and laboratory findings were compatible with acute appendicitis and the surgical removal was decided. The resected appendix measured 7 × 2.2 cm, with a congested and inflamed wall filled of stercolith (Fig. 1). Histological examination of the hematoxylin–eosin-saffron (HES) stained sections of the resected appendix showed intense acute inflammation pattern of the appendiceal wall with numerous neutrophils, plasma cells, and macrophages as well as lymphoid hyperplasia, epithelial ulceration and suppurative necrosis; the lumen of the appendix contained numerous adult parasites with histological characteristic features of *E. vermicularis* (Figs. 2a, 3, black arrows). The cross-section of the parasites showed the two lateral cuticular projections (alae) extending from the thin eosinophilic wall (Fig. 3a, b, black arrows). In the lumen of some female parasites, many oval eggs were seen in their gravid genital tract (Fig. 3a, red arrows). A male parasite was also seen with its characteristic testicular structures (Fig. 3b, red arrow).

These histological findings were typical of acute *E. vermicularis* appendicitis. The patient recovered well after surgery and mebendazole has been prescribed for him as well as his close family members.

## Discussion and conclusion

*E. vermicularis* belongs to the group of nematodes, humans are the only known natural hosts of this parasite [5, 6]. Humans get infection by ingesting eggs from contaminated food, water, dust or even by hand contacts from the infected persons [5]. Embryonated eggs measure from 30 to 60 µm, hatch in the stomach of the new infected patients, and transform into larvae that migrate to caecum where they mature into adults pinworms that approximately measure 1 cm in length [6]. The caecal region constitutes the common habitat of adult parasites where they live and mate. At night, the gravid female travel to the anal region to deposit thousands of eggs. These eggs can lead to a new infection if ingested at least 6 h after being laid [3, 6]. The female parasite is very mobile while depositing eggs at night in the anal region, leading to a harsh nocturnal pruritus that can cause insomnia, loss of appetite, loss of weight, especially in children. Eggs are eliminated in the faeces of infected patients, or they stick under finger nails when the patient scratches his anus. A new cycle of infection can resume when another person ingests food or water containing eggs; usually an auto-infestation occurs as the infected patients get the parasite by ingesting eggs from their fingers and hands.

Usually, the pathogenicity of *E. vermicularis* is mild, ranging from asymptomatic to nocturnal anal pruritus [2]. In fact, our patient did not report any clinical symptoms suggestive of oxyuriasis, such as nocturnal anal itching. However, cases of patients with symptoms of clinical acute appendicitis associated with *E. vermicularis* have been reported in the literature [7]. The reported incidence of *E. vermicularis* appendicitis varies widely from 0.2 to 41.8%, and young girls are mostly affected [2, 10]. Our current case is epidemiologically particular as it was an adult male patient. However controversies and speculations still remain in regard to the pathophysiology of appendicitis associated with *E. vermicularis* [3]. Several studies support the fact that clinical symptoms of appendicitis seem to result from luminal obstruction by adult parasites, rather than a true inflammation of the appendiceal wall [1, 6–8, 11]. When located in the appendix lumen, the parasite causes contraction of the appendiceal wall (appendiceal colic) leading to clinical symptoms similar to those of the classic appendicitis [1]. In fact, mostly resected appendiceal specimens from these patients showed no evidence of histological inflammation [7, 8, 11]. In a recent study by Lala and Upadhyay [7], of 2923 resected appendices in pediatric population, only 4% showed *E. vermicularis* on histology, and only 25% of these parasitic appendicitis showed concurrent histological acute inflammation. The common histological findings in resected appendiceal specimens range from normal to various inflammatory patterns such as lymphoid hyperplasia, eosinophilic infiltrate, or neutrophilic infiltrate [1, 3, 6]. In our case, intense acute inflammatory patterns were observed: mucosal ulceration, suppurative necrosis with numerous neutrophils and plasma cells as well as lymphoid hyperplasia in the appendiceal wall. On macroscopic examination, stercoliths were seen in the appendix wall, but the parasites were not seen. In contrast, some authors have reported macroscopically visible parasites [2]. In our case, what is questionable, is the role of the parasites found in the specimen. Are they the causative agents of the acute suppurative appendicitis or an incidental finding? Perhaps the appendicitis resulted from the luminal obstruction by stercoliths found on macroscopic examination.

Also, aberrant migrations leading to extraintestinal findings of *E. vermicularis* have been reported, in the liver [12], bladder [13], lungs [14], kidney [15] or in the female genital tract [16, 17]. Thus, this parasite can have a wide range of pathogenicity, implying that pathologists should be familiar to its histological aspects for correct diagnosis. In fact, the histologic identification of *E. vermicularis* or its eggs on resected specimens is not difficult when the pathologist performs a careful searching for this parasite. In appendiceal specimens, the parasites are mostly evident in the lumen. The cross section shows the characteristic double and lateral “thorn-like” extensions (alae) from the thin eosinophilic wall of the parasite [3]. Within the wall of parasites, annular structures are easily seen, corresponding to the intestine, also numerous oval eggs, with flattened edges can be found in gravid females [3, 18, 19]. Adult male parasites look smaller than adult females, they are recognisable by their characteristic genitals with finely granular and round-shaped appearance [18]. However in solid organs like ovaries, spleen or liver, the histological diagnosis can be tricky as parasites have no sufficient space to develop. Granulomas and other inflammatory changes around oval structures (eggs) can be the main aspects to lead the pathologist to the correct diagnosis.

In summary, histological patterns of acute appendicitis associated with *E. vermicularis* are a very rare finding. Care should be taken by pathologists to thoroughly examine any appendiceal specimen to search for this parasite for appropriate anti-helminthic treatment of patients and their close family members.

## Abbreviations

*E. vermicularis*: *Enterobius vermicularis*
HES: hematoxylin–eosin-saffron

## References

[1] Aydin O (2007). Incidental parasitic infestations in surgically removed appendices: a retrospective analysis. Diagn Pathol..

[2] Ariyarathenam AV, Nachimuthu S, Tang TY, Courtney ED, Harris SA, Harris AM (2010). *Enterobius vermicularis* infestation of the appendix and management at the time of laparoscopic appendectomy: case series and literature review. Int J Surg..

[3] Lamps LW (2010). Infectious causes of appendicitis. Infect Dis Clin N Am..

[4] Gunawardena NK, Chandrasena TN, de Silva NR (2013). Prevalence of enterobiasis among primary school children in Ragama, Sri Lanka. Ceylon Med J..

[5] Salim N, Schindler T, Abdul U, Rothen J, Genton B, Lweno O (2014). Enterobiasis and strongyloidiasis and associated co-infections and morbidity markers in infants, preschool- and school-aged children from rural coastal Tanzania: a cross-sectional study. BMC Infect Dis.

[6] Panidis S, Paramythiotis D, Panagiotou D, Batsis G, Salonikidis S (2011). Acute appendicitis secondary to *Enterobius vermicularis* infection in a middle-aged man: a case report. J Med Case Rep..

[7] Lala S, Upadhyay V (2016). *Enterobius vermicularis* and its role in paediatric appendicitis: protection or predisposition?. ANZ J Surg.

[8] Akkapulu N, Abdullazade S (2016). Is *Enterobius vermicularis* infestation associated with acute appendicitis?. Eur J Trauma Emerg Surg.

[9] Hamdona SM, Lubbad AM, Al-Hindi AI (2016). Histopathological study of *Enterobius vermicularis* among appendicitis patients in Gaza strip, Palestine. J Parasit Dis..

[10] Ahmed MU, Bilal M, Anis K, Khan AM, Fatima K, Ahmed I (2015). The frequency of *Enterobius vermicularis* infections in patients diagnosed with acute appendicitis in Pakistan. Glob J Health Sci..

[11] Sodergren MH, Jethwa P, Wilkinson S, Kerwat R (2009). Presenting features of *Enterobius vermicularis* in the vermiform appendix. Scand J Gastroenterol.

[12] Arkoulis N, Zerbinis H, Simatos G, Nisiotis A (2012). *Enterobius vermicularis* (pinworm) infection of the liver mimicking malignancy: presentation of a new case and review of current literature. Int J Surg Case Rep..

[13] Sammour ZM, Gomes CM, Tome AL, Bruschini H, Srougi M (2008). Prolonged irritative voiding symptoms due to *Enterobius vermicularis* bladder infestation in an adult patient. Braz J Infect Dis..

[14] Beaver PC, Kriz JJ, Lau TJ (1973). Pulmonary nodule caused by *Enterobius vermicularis*. Am J Trop Med Hyg.

[15] Cateau E, Yacoub M, Tavilien C, Becq-Giraudon B, Rodier MH (2010). *Enterobius vermicularis* in the kidney: an unusual location. J Med Microbiol.

[16] Powell G, Sarmah P, Sethi B, Ganesan R (2013). *Enterobius vermicularis* infection of the ovary. BMJ Case Rep..

[17] Ngui R, Ravindran S, Ong DB, Chow TK, Low KP, Nureena ZS (2014). *Enterobius vermicularis* salpingitis seen in the setting of ectopic pregnancy in a Malaysian patient. J Clin Microbiol.

[18] https://www.cdc.gov/dpdx/enterobiasis/gallery.html#tissue. Accessed 10 Dec 2016.

[19] Lauwers G, Mino-Kenudson M, Kradin RL, Kradin Richard L (2010). Infections of the gastrointestinal tract. Diagnostic pathology of infectious disease.

